# Open versus laparoscopic repair of inguinal hernia: an overview of systematic reviews of randomised controlled trials

**DOI:** 10.1007/s00464-022-09161-6

**Published:** 2022-03-14

**Authors:** Nafi’u Haladu, Adegoke Alabi, Miriam Brazzelli, Mari Imamura, Irfan Ahmed, George Ramsay, Neil W. Scott

**Affiliations:** 1 Institute of Applied Health Sciences, University of Aberdeen, Aberdeen, UK; 2 Emergency Department, Southend University Teaching Hospital, Westcliff-on-Sea, UK; 3 Luton and Dunstable University Hospital, Luton, UK; 4 Health Services Research Unit, University of Aberdeen, Aberdeen, UK; 5 Department of Surgery, NHS Grampian, Aberdeen, UK; 6 Medical Statistics Team, University of Aberdeen, Polwarth Building, Foresterhill, Aberdeen, AB25 2ZD UK

**Keywords:** Inguinal hernia surgery, Laparoscopic repair, Open repair, Primary hernia, Recurrent hernia, Overview of systematic reviews

## Abstract

**Background:**

Inguinal hernia has a lifetime incidence of 27% in men and 3% in women. Surgery is the recommended treatment, but there is no consensus on the best method. Open repair is most popular, but there are concerns about the risk of chronic groin pain. Laparoscopic repair is increasingly accepted due to the lower risk of chronic pain, although its recurrence rate is still unclear. The aim of this overview is to compare the risk of recurrence and chronic groin pain in laparoscopic versus open repair for inguinal hernia.

**Methods:**

We searched Ovid MEDLINE, EMBASE and the Cochrane Database of Systematic Reviews for systematic reviews and meta-analyses. Only reviews of randomised controlled trials (RCTs) in adults published in English were included. Conference proceedings and editorials were excluded. The quality of the systematic reviews was assessed using the AMSTAR 2 checklist. Two outcomes were considered: hernia recurrence and chronic pain.

**Results:**

Twenty-one systematic reviews and meta-analyses were included. Laparoscopic repair was associated with a lower risk of chronic groin pain compared with open repair. In the four systematic reviews assessing any laparoscopic versus any open repairs, laparoscopic repair was associated with a statistically significant (range: 26–46%) reduction in the odds or risk of chronic pain. Most reviews showed no difference in recurrence rates between laparoscopic and open repairs, regardless of the types of repair considered or the types of hernia that were studied, but most reviews had wide confidence intervals and we cannot rule out clinically important effects favouring either type of repair.

**Conclusion:**

Meta-analyses suggest that laparoscopic repairs have a lower incidence of chronic groin pain than open repair, but there is no evidence of differences in recurrence rates between laparoscopic and open repairs.

Inguinal hernia accounts for 75% of all abdominal wall hernias and has a lifetime incidence of 27% in males and 3% in women [1]. Several types of inguinal hernia have been identified and surgery to repair them, which began around the sixteenth century following the establishment of modern anatomy, has since evolved with a number of techniques currently available [2]. There has been ongoing debate about which form of repair offers the best patient outcomes and there is yet to be a unanimously agreed superior approach to the management of inguinal hernias.

Open inguinal hernia repair has long been the method of choice for most surgeons and is often recommended in contemporary literature as the optimal approach for primary unilateral inguinal hernia, which is a hernia occurring for the first time on one side of the groin, without any prior repair [3, 4]. Open repairs have mainly been classified as open mesh (e.g. Lichtenstein) or open non-mesh (e.g. Shouldice) repairs based on whether a synthetic material has been used to re-enforce the repaired posterior wall [5]. Tension-free mesh repair (Lichtenstein technique) is usually considered the repair method of choice among open repairs due to its easy reproducibility by non-specialist surgeons. However, there are concerns about the risk of chronic groin pain, although recurrence rates have been noticeably very low [6].

Trans-abdominal pre-peritoneal repair (TAPP) and the totally extra-peritoneal repair (TEP) are two of the main laparoscopic (keyhole) techniques used. Laparoscopic approaches have grown in popularity recently with some surgeons appreciative of its significantly lower incidence of long-term post-operatively pain, but there have been some concerns regarding a possible increased risk of recurrence after TEP repair [7]. This has been reported more frequently in primary, unilateral inguinal hernia compared with recurrent hernia. Despite this concern, TEP has nonetheless been adopted as the procedure of choice because of a lower risk of intra-abdominal injuries compared to TAPP repair as well as the comparably good outcomes especially when it is done by skilled surgeons [3, 4, 7–10].

Several systematic reviews and meta-analyses have compared laparoscopic and open repair techniques but there has not been any consensus on which technique offers better outcomes overall. This study aims to conduct an overview of existing systematic reviews, which is a relatively new methodology for summarising evidence. Compared with conducting a new systematic review, an overview takes considerably less time and resources and can help researchers synthesise evidence across interventions, especially where conflicting evidence has been reported from existing systematic reviews, with the aim of establishing a comprehensive overview on the current best evidence [11].

This overview of reviews aims at informing clinical practice by identifying, analysing and synthesising the numerous published systematic reviews assessing the comparative efficacy of open and laparoscopic repairs for inguinal hernia.

## Materials and methods

### Study design and protocol

An overview of systematic reviews was conducted according to the recommendations of the Cochrane Handbook of Systematic Reviews of Interventions to gain understanding of the currently available evidence for the efficacy of inguinal hernia repairs from existing systematic reviews in the literature [11]. The method of the overview was pre-specified in a research protocol based on the PRISMA reporting guidelines [12].

### Types of reviews

This overview included systematic reviews (including meta-analyses and network meta-analyses (NMA)) of randomised controlled trials (RCTs) published in English. Systematic reviews that included a mixture of randomised and non-randomised evidence were included if they reported RCT data separately. Conference proceedings, protocols and editorials were excluded.

### Types of participants

Males and females aged 16 years or above.

### Types of interventions

Laparoscopic surgery was compared with open surgery for the repair of inguinal hernias. When possible, we also included studies assessing TAPP and/or TEP repairs separately. Similarly, we included studies assessing all open repairs as well as open mesh and non-mesh repairs separately.

### Outcomes

Hernia recurrence and incidence of chronic groin pain.

### Literature search

We searched Ovid MEDLINE, EMBASE and CDSR (Cochrane Database of Systematic Reviews) to identify systematic reviews and meta-analyses published up to 8 May 2020. There were no restrictions on date or language of publication. An initial search strategy was generated for Ovid MEDLINE and adapted to other databases. The search focused on free-text and MeSH terms for ‘inguinal hernia surgery’, ‘open repair techniques’, ‘laparoscopic repair techniques’ and ‘systematic reviews and meta-analysis’. Additionally, we checked the reference lists of retrieved reviews for additional eligible reviews.

### Screening and study selection

Two researchers (NH, AA) searched for and selected reviews based on criteria pre-specified in the research protocol. Initially, the researchers screened the titles and abstracts identified by the search strategy independently. Disagreements were resolved by discussion or arbitration by other authors. Selected studies were retrieved in full and assessed for inclusion by one author (NH).

### Data collection

A data extraction form was developed and piloted to record relevant data from the identified systematic reviews. Recorded data included administrative data, bibliographic information, descriptive characteristics of reviews including inclusion/exclusion criteria, information on the type of hernia and definitions of the outcomes. Data on hernia recurrence and chronic pain were extracted by one researcher (NH) in the form of effect sizes and their 95% confidence intervals (or credible intervals for NMA).

### Quality assessment of included reviews

The quality of included reviews was assessed using the AMSTAR 2 checklist, a 16-item tool that has been developed to appraise the methodological quality of overviews of systematic reviews [13]. AMSTAR 2 classifies overall confidence in the results of each review as ‘high’, ‘moderate’, ‘low’ or ‘critically low’ based on ratings for selected items identified as the critical domains. The items considered critical domains for the purpose of this overview included ‘duplicate study selection by review authors’ (item 5), ‘adequate description of included studies in the review’ (item 8), ‘use of appropriate method for meta-analysis’ (item 11) and either of ‘use of a satisfactory risk of bias (ROB) assessment for included studies in the review’ (item 9) or ‘assessment of the impact of ROB in interpretation of results’ (item 13).

#### Data synthesis

Quantitative outcome data were summarised in tables showing effect sizes and 95% confidence/credible intervals for hernia recurrence and chronic groin pain, bearing in mind that results of the reviews may include overlapping studies. Where no results from meta-analysis were available, the reported conclusions were included in the tables. A narrative synthesis was then carried out.

## Results

### Literature search

**Fig. 1:**
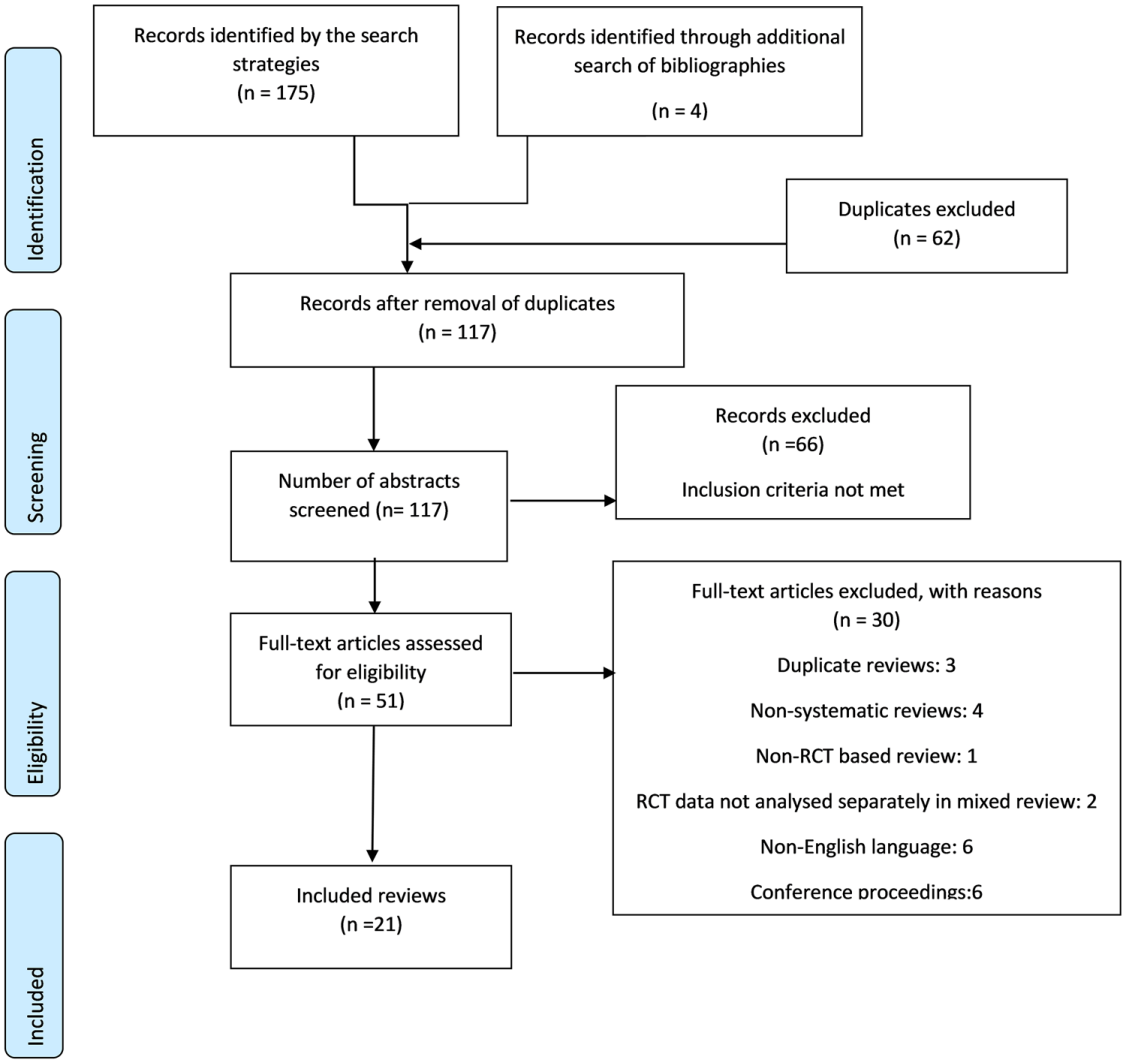
PRISMA flow diagram

The initial search retrieved 175 systematic reviews with an additional four reviews identified from the reference lists of the included reviews. During abstract screening, sixty-two duplicate reviews were excluded, and a further 66 reviews failed to meet the pre-specified inclusion criteria. After full-text screening of the remaining 51 articles, 21 reviews met the inclusion criteria (Fig. 1).

### Study details

Details of the study characteristics are summarised in Table 1. The included reviews were published between 1999 and 2020. The most recent search date for an included review was February 2019. The number of RCTs per review varied from 4 to 58, while the number of participants ranged from 404 to 17,510. The age of the participants ranged from 16 to 100 years. All the reviews included participants of both genders. Five reviews [8, 21, 27, 29, 32] analysed only data on hernia recurrence and did not report chronic pain while the remaining 16 reviews [10, 14–20, 22–26, 28, 30, 31] reported data on both outcomes. Five reviews [15, 16, 19, 23, 25] compared outcomes in primary inguinal hernias only, and five reviews [20, 21, 24, 27, 28] examined recurrent inguinal hernias alone, while the remaining 11 reviews [8, 10, 14, 17, 18, 22, 26, 29–32] either considered both types of hernia or did not specify the type. One Cochrane review was identified which collected individual participant data (IPD) from the trialists which enabled reporting of separate subgroups for primary and recurrent hernias [10]. One NMA was also identified [15].

**Table 1:** Characteristics of included reviews

Review ID	Search dates	No. of studies analysed	No. of participants	Gender *All/M/F*	Age in years (study policy)*	Hernia types		Comparison
						Primary or Recurrent	Unilateral or Bilateral	
Lyu [14]	Up to Sept. 2018	31	5594	All	25–69	PH, RH	UH, BH	TEP vs Lichtenstein, TAPP vs Lichtenstein
Aiolfi 2019 [15]	2000 to Feb. 2019	12	3748	All	18–76	PH	UH	TEP vs Open, TAPP vs Open
Bullen 2019 [16]	Up to April 2019	12	3966	All	NR (≥ 16)	PH	UH	Lap vs Lichtenstein
Gavrilidis 2019 [17]	1989 to 2019	21	6573	NR	36–68	NR	NR	TEP vs Lichtenstein
Patterson 2019 [18]	Jan 1998 to May 2018	58	17,510	All	23.6—65.4	NR	NR	Lap vs Open, TAPP vs Lichtenstein, TAPP vs Shouldice, TEP vs Lichtenstein, TEP vs Shouldice
Scheuermann 2017 [19]	Up to July 2016	8	896	All	19–84	PH	NR	TAPP vs Lichtenstein
Pisanu 2015 [20]	1966 to 2013	7	688	All	42–75	RH	NR	Lap vs Lichtenstein
Li 2014 [21]^+^	Jan 1999 to Sept 2012	6 (11)	643 (1311)	NR	NR	RH	NR	Lap vs Open
Zheng 2014 [22]	Up to Jan 2013	13	3279	All	NR (18–100)	PH, RH	NR	TEP vs Lichtenstein
Koning 2013 [23]	1966 to Jan 2012	13	5404	All	NR (Adults)	PH	UH, BH	TEP vs Lichtenstein
Yang 2013 [24]	1966 to May 2012	5	429	NR	52–66	RH	NR	Lap vs Lichtenstein
O’Reily 2012 [25]	Up to March 2012	27	7161	NR	35.6—65.5	PH	UH	Lap vs Open, TEP vs Open, TAPP vs Open
Aly 2011 [26]	1995 to May 2010	8	5300	NR	16–85	NR	NR	Lap vs Open
Dedemadi 2010 [27]	1990 to 2008	7	663	All	55.8–64	RH	NR	TEP vs Lichtenstein, Lap vs Lichtenstein
Karthikesalingam 2010 [28]	1966 to 2009	4	404	NR	NR	RH	NR	Lap vs Open
Kuhry 2007 [29]	NR	23	4231	NR	NR	NR	NR	TEP vs Open
Schmedt 2005 [30]	Up to April 2014	34	7223	NR	NR	NR	NR	Lap vs Lichtenstein
McCormack 2003 [10]	NR	41	7161	All	NR (= / > 18)	PH, RH	UH, BH	Lap vs Open, TEP vs Open, TAPP vs Open
Memon 2003 [8]	Jan 1990 to Oct 2000	29	5588	NR	NR	NR	NR	Lap vs Open, TEP vs Open, TAPP vs Open
Schmedt 2002 [31]	NR	33	5053	NR	NR	NR	NR	Lap vs Lichtenstein, Lap vs Shouldice
Chung 1999 [32]	May 1994 to March 1997	14	2471	NR	NR	NR	NR	Lap vs Shouldice, TAPP vs Lichtenstein

*NR* not reported, *PH* Primary inguinal hernia, *RH* Recurrent inguinal hernia, *UH* Unilateral hernia, *BH* Bilateral hernia, *TAPP* Transabdominal pre-peritoneal repair, *TEP* Totally extra-peritoneal repair, *Lap* Laparoscopic procedure, * Age range normally reported but where not available, the age policy for review noted in brackets, + Mixed method studies with No. of RCTs/participants displayed and total no. of studies/participants in brackets

### Methodological quality of included reviews

Two reviews [15, 16] were scored as high quality (both published in 2019) (Table 2). Nine studies were judged to be of moderate quality [10, 14, 17–21, 23, 28]. Among the six older studies (before 2010), only the Cochrane review [10] was found to be of moderate quality and there were three reviews [29, 31, 32] of critically low quality.

**Table 2:** Summary of risk of bias for included reviews (AMSTAR 2 Checklist)

Review ID	1	2	3	4	5	6	7	8	9	10	11	12	13	14	15	16	Overall confidence rating
Lyu 2020^14^	Y	PY	N	PY	Y	Y	PY	PY	Y	N	Y	N	Y	Y	N	Y	Moderate
Aiolfi 2019^15^	Y	Y	Y	PY	Y	Y	PY	Y	Y	N	Y	Y	Y	Y	N	Y	High
Bullen 2019^16^	Y	Y	N	Y	Y	Y	PY	Y	Y	N	Y	Y	Y	Y	Y	Y	High
Gavrilidis 2019^17^	Y	Y	N	Y	Y	Y	PY	Y	Y	N	Y	Y	Y	Y	N	Y	Moderate
Patterson 2019^18^	Y	PY	N	Y	Y	Y	PY	PY	Y	N	Y	Y	Y	Y	N	Y	Moderate
Scheuermann 2017^19^	Y	Y	Y	Y	N	N	PY	Y	Y	N	Y	Y	N	Y	Y	Y	Moderate
Pisanu 2015^20^	Y	Y	N	Y	Y	Y	PY	PY	Y	N	Y	Y	Y	Y	N	Y	Moderate
Li 2014 ^21^	Y	N	N	PY	Y	Y	PY	Y	Y	N	Y	Y	N	Y	Y	Y	Moderate
Zheng 2014^22^	Y	N	N	PY	N	Y	PY	Y	Y	N	Y	Y	N	Y	N	Y	Low
Koning 2013^23^	Y	Y	N	PY	Y	Y	PY	Y	Y	N	Y	Y	Y	Y	N	Y	Moderate
Yang 2013^24^	Y	N	N	Y	Y	Y	PY	Y	Y	N	Y	N	N	Y	N	Y	Low
O’Reily 2012^25^	Y	N	N	PY	N	N	PY	Y	Y	N	Y	Y	Y	Y	N	Y	Low
Aly 2011^26^	Y	N	N	Y	N	N	N	Y	Y	N	Y	Y	Y	Y	N	N	Low
Dedemadi 2010^27^	Y	N	N	Y	Y	Y	N	Y	Y	N	Y	Y	Y	Y	Y	N	Low
Karthikesalingam 2010^28^	Y	N	N	Y	Y	Y	PY	Y	Y	N	Y	Y	Y	Y	Y	Y	Moderate
Kuhry 2007^29^	Y	N	N	PY	N	N	N	PY	N	N	NA	NA	N	Y	NA	N	Critically low
Schmedt 2005^30^	Y	N	N	N	N	N	Y	Y	PY	N	Y	Y	N	Y	N	N	Low
McCormack 2003^10^	Y	Y	N	Y	Y	Y	Y	Y	Y	N	Y	Y	Y	Y	N	Y	Moderate
Memon 2003^8^	Y	N	N	PY	Y	Y	N	PY	Y	N	Y	Y	Y	Y	N	N	Low
Schmedt 2002^31^	Y	N	N	N	N	N	N	Y	N	N	NA	NA	N	Y	NA	N	Critically low
Chung 1999^32^	Y	N	N	N	N	N	N	Y	N	N	Y	N	N	N	N	N	Critically low

1-Participants, interventions, comparators, outcomes (PICO) components; 2- Predetermined protocol; 3-Explanation for study design choice; 4-Comprehensive literature search; 5-Duplicate study selection; 6-Duplicate data extraction; 7-List of excluded studies; 8-Description of included studies; 9-Satisfactory risk of bias (ROB) assessment for included studies in the review; 10- Funding sources for included studies in the review; 11- Appropriate method for meta-analysis; 12-Impact of study ROB on results; 13- Impact of ROB in interpretation of results; 14- Explanation for heterogeneity in results; 15- Publication bias; 16- Conflicts of interest; Y- Yes; N- No; PY- Partial yes; NA- not applicable

### Hernia recurrence

Table 3 presents the results for hernia recurrence, first for laparoscopic versus open overall and then for specific combinations of repairs. Results for primary and recurrent hernias are provided in separate columns where available, although usually the first of these columns represents a mixture of primary and recurrent hernias or a situation where it was unclear which types were included. Overall, most reviews showed no evidence of differences in recurrence rates between laparoscopic and open repairs, regardless of the type of hernia studied. However, most of the reviews had wide confidence intervals (CIs), so we cannot rule out clinically important effects favouring either laparoscopic or open repair.

**Table 3:** Results for Hernia recurrence (OR/RR > 1 or RD > 0 favours Open/Open mesh/Open non-mesh repairs)

Review ID	Type(s) of Hernia	Effect measure	Primary or mixed hernia			Recurrent hernia		Study quality (AMS-TAR2)*	Comments
			Recurrence rates	No. of studies (no. of participants)	Effect size (95% CI/CrI)	No. of studies (no. of participants)	Effect size (95% CI/CrI)		
*Laparoscopic vs open*									
Patterson 2019 [18]	All	RR	Lap (4.4%) Open (3.9%)	46 (15,605)	0.94 (0⋅72, 1.24)			M	Included only studies published after Jan 1998
McCormack 2003 [10]	All	Peto OR	Lap 86/3138 (2.7%) Open 109/3504 (3.1%)	27 (6642)	0.81 (0.61, 1.08)	11 (387)	1.04 (0.45, 2.43)	M	
O’Reilly 2012 [25]	PH, UH	RR	NR	18 (6874)	2.06 (1.26, 3.37)			L	
Li 2014 [21]	RH	RD				6 (643)	− 0.01 (− 0.06, 0.03)	M	
Kerthakesalingam 2010 [28]	RH	OR				4 (NR)	0·84 (0·33, 2·17)	M	
Aly 2011 [26]	NR	OR	Lap 125/2047 (6.1%) Open 59/2079 (2.8%)	6 (4126)	2.17 (1.58, 2.98)			L	
Memon 2003 [8]	NR	OR	Lap 89/2864 (3.1%) Open 81/2818 (2.9%)	20 (5682)	1·51 (0·81, 2·79)			L	
*Laparoscopic vs Open mesh*									
Bullen 2019 [16]	PH, UH	OR	Lap 55/1865 (2.9%) Open 45/1758 (2.6%)	9 (3623)	1.14 (0.51, 2.55)			H	Included studies with follow-up duration up to 12 months
Pisanu 2015 [20]	RH	OR				5 (534)	0.67 (0.39, 1.16)	M	
Yang 2013 [24]	RH	OR				5 (427)	0.68 (0.33, 1.41)	L	
Dedemadi 2010 [27]	RH	RR				7 (706)	0.72 (0.45, 1.14)	L	
Schmedt 2005 [30]	NR	Peto OR	Lap 112/2042 (5.5%) Open 56/2058 (2.7%)	14 (4100)	2.00 (1.46, 2.74)			L	Excluded studies that considered IPOM as endoscopic procedure
Schmedt 2002 [31]^#^	NR	NR		11 (3482)	NR			CL	No significant difference reported
*Laparoscopic vs Open non-mesh*									
Schmedt 2002 [31]^#^	NR	NR		18 (2700)	NR			CL	No significant difference reported
Chung 1999 [32]	NR	OR	NR	6 (1711)	-1.48 (-4.31, 1.34)			CL	Negative values for odds ratios were reported and it’s unclear what this effect size represents
*TAPP vs open*									
McCormack 2003 [10]	All	Peto OR	Lap 50/1763 (2.8%) Open 71/2126 (3.3%)	27 (3889)	0.76 (0.52, 1.09)	10 (276)	0.99 (0.39, 2.51)	M	IPD
Aiolfi 2019 [15]	PH, UH	RR	NR	NR	0.96 (0.57, 1.51)			H	NMA
O’Reilly 2012 [25]	PH, UH	RR	NR	11 (2656)	1.14 (0.78, 1.68)			L	
Memon 2003 [8]	NR	OR	Lap 54/1985 (2.7%) Open 41/1904 (2.2%)	15 (3862)	1·52 (0·68, 3·41)			L	
*TAPP vs open mesh*									
Lyu 2020 [14]	All	OR	NR	NR	1.7 (0.56, 5.5)			M	NMA
Patterson 2019 [18]	All	RR	NR	12 (NR)	0⋅79 (0⋅52, 1⋅21)		NR	M	
McCormack 2003 [10]	All	Peto OR	Lap 22/912 (2.4%) Open 21/918 (2.3%)	12 (1830)	1.01 (0.56, 1.85)	5 (190)	1.20 (0.43, 3.32)		IPD
Scheuermann 2017 [19]	PH	OR	Lap 9/337 (2.7%) Open 6/322 (1.9%)	6 (659)	1.17 (0.39, 3.57)			M	Excluded irreducible and incarcerated hernia requiring emergency surgery
Chung 1999 [32]	NR	OR	NR	6 (607)	0.00 (-0.16, 0.16)			CL	
*TAPP vs open non-mesh*									
Patterson 2019 [18]	All	RR	NR	8 (NR)	0⋅96 (0⋅69, 1⋅33)			M	Included only studies published after Jan 1998
McCormack 2003 [10]	All	Peto OR	Lap 26/1140 (2.3%) Open 47/1119 (4.2%)	16 (2259)	0.45 (0.28, 0.72)	4 (93)	0.31 (0.04, 2.26)	M	IPD
*TEP vs open*									
McCormack 2003 [10]	All	Peto OR	Lap 34/1375 (2.5%) Open 38/1378 (2.8%)	7 (2753)	0.91 (0.57, 1.46)	2 (111)	1.33 (0.18, 10.06)	M	IPD
Aiolfi 2019 [15]	PH, UH	RR	NR	NR	1.0 (0.65, 1.61)			H	NMA
O’Reilly 2012 [25]	PH, UH	RR	NR	10 (3063)	3.72 (1.66, 8.35)			L	
Kuhry 2007 [29]^#^	NR	NR		15 (2937)	NR			CL	1 of 15 studies showed statistically significant lower recurrence in TEP, others reported no difference
Memon 2003 [8]	NR	OR	Lap 25/856 (2.9%) Open 36/887 (4.1%)	4 (1743)	0·98 (0·35, 2·70)			L	
*TEP vs open mesh*									
Lyu 2020 [14]	All	OR	NR	NR	0.85 (0.26, 2.0)			M	NMA
Patterson 2019 [18]	All	RR	NR	14 (NR)	1⋅05 (0⋅58, 1⋅91)			M	Included only studies published after Jan 1998
McCormack 2003 [10]	All	Peto OR	Lap 7/351 (2.0%) Open 7/327 (2.1%)	6 (678)	0.97 (0.34, 2.77)	1 (36)	0.23 (0.01, 4.48)		IPD
Zheng 2014 [22]	All	RR	Lap 45/1645 (2.7%) Open 29/1742 (1.7%)	9 (3387)	1.64 (1.05, 2.55)			L	
Koning 2013 [23]	PH	RR	Lap 130/2582 (5.0%) Open 69/2598 (2.3%)	12 (5180)	1.89 (1.42, 2.50)			M	
Dedemadi 2010 [27]	RH	RR				4 (344)	0.48 (0.18, 1.33)	L	
Gavrilidis 2019 [17]	NR	Peto OR	Lap 149/2678 (5.6%) Open 99/2790 (3.5%)	14 (5468)	1.58 (1.22, 2.04)			M	
*TEP vs open non-mesh*									
Patterson 2019 [18]	All	OR	NR	2 (NR)	1⋅73 (0⋅07, 40⋅38)		NR	M	Included only studies published after Jan 1998
McCormack 2003[10]	All	Peto OR	Lap 20/739 (2.7%) Open 31/780 (4.0%)	5 (1519)	0.67 (0.38, 1.18)			M	IPD

^*^H high, *M* moderate, *L* low, *CL* critically low

^#^ Review using qualitative synthesis and no meta-analysis

*All* includes both primary and recurrent inguinal hernias (PH and RH) and unilateral and bilateral inguinal hernias (UH and BH), *BH* Bilateral inguinal hernia, *CI* confidence interval, *CrI* credible interval, *IPD* used individual participant data, *Lap* laparoscopic, *NMA* network meta-analysis, *NR* not reported/no meta-analysis done, *OR* odds ratio, *Peto OR* Peto odds ratio, *PH* primary inguinal hernia, *RCT* randomised controlled trials, *RD* risk difference, *RH* recurrent inguinal hernia, *RR* relative risk, *TAPP* transabdominal pre-peritoneal repair, *TEP* totally extra-peritoneal repair, *UH* unilateral inguinal hernia

Six reviews [10, 15, 16, 19, 23, 25] presented data for primary inguinal hernias alone and most reported no statistically significant differences between laparoscopic and open repairs. One review [25] showed lower recurrence for the open group versus both laparoscopic [RR 2.06 (1.26, 3.37)] and TEP groups [RR 3.72 (1.66, 8.35)]. However, this review included fewer studies compared with earlier published reviews. Similarly, six reviews [10, 20, 21, 24, 27, 28] presented results specifically for recurrent hernias and none reported statistically significant differences between laparoscopic and open groups.

Seven reviews [8, 17, 26, 29–32] did not report the specific hernia types they considered and were assumed to have included both primary and recurrent hernias (Table 3). These reviews generally reported considerable uncertainty in the magnitude and direction of their effects. Two [17, 26] of these reviews reported findings in favour of open repair techniques and one [26] showed a doubling of the odds of recurrence after laparoscopic repair [OR 2.17 (1.58, 2.98)]. However, it considered only six studies, significantly fewer than those included in earlier published reviews.

When comparing specific types of laparoscopic repair, most reviews comparing TAPP with open repair showed no statistically significant results. One study which used individual participant data (IPD) [10] found a lower risk of recurrence for TAPP versus open non-mesh repair, but this finding was not replicated in a more recent review [18].

When comparing TEP versus open repair, results of the meta-analyses varied considerably and most had wide confidence intervals. Three reviews [17, 22, 25] found evidence of fewer recurrences for open repairs compared with TEP.

### Chronic pain

Definitions of chronic pain varied and ranged from one month to one year after the procedure. Five reviews [15, 16, 19, 23, 25] reporting this outcome studied primary inguinal hernias alone and another five [20, 21, 24, 27, 28] looked at only recurrent inguinal hernias (Table 4).

**Table 4:** Results for Chronic pain (OR/RR > 1 or RD > 0 favours Open/Open mesh/Open non-mesh repairs)

Review ID				Primary and mixed hernia			Recurrent hernia			Comments
	Type of Hernia	Review’s definition of chronic pain	Effect measure	Chronic pain rates	No. of studies (no. of participants)	Effect size (95% CI/CrI)	No. of studies (no. of participants)	Effect size (95% CI/CrI)	Study quality (AMS-TAR2)*	
*Laparoscopic vs Open*										
Patterson 2019 [18]	All	Pain between 6 months and up to 1 year	RR	Lap 350/2999 (11.7%) Open 485/3133 (15.5%)	17 (6132)	0.74 (0⋅59, 0.93)			M	Included only studies published after Jan 1998
	All	Pain after 1 year	RR	Lap 314/4290 (7.3%) Open 537/4481 (12.0%)	19 (8771)	0.62 (0⋅47, 0⋅82)			M	
McCormack 2003 [10]	All	Groin pain of any severity as near 12 months after the operation as possible provided this was at least after 3 months	Peto OR	Lap 290/2101 (13.8%) Open 459/2399 (19.1%)	21 (749)	0.54 (0.46, 0.64)	8 (331)	0.90 (0.50, 1.59)	M	IPD
O’Reilly 2012 [25]	PH, UH	Persistence of symptoms beyond 1 month	RR	NR	13 (4209)	0.66 (0.51, 0.87)			L	
Kerthakesalingam 2010 [28]	RH	Severe chronic pain after at least 1 year	OR				3 (NR)	0·91 (0·14, 5·88)	M	
*Laparoscopic vs Open mesh*										
Bullen 2019 [16]	PH	Pain lasting more than 6 months	OR	Lap 168/1780 (9.4%) Open 343/1665 (20.6%)	10 (3445)	0.41 (0.30, 0.56)			H	
Pisanu 2015 [20]	RH	NR	OR				4 (377)	0.39 (0.21, 0.72)	M	
Yang 2013 [24]	RH	NR	Peto OR				4 (377)	0.33 (0.17, 0.68)	L	
Schmedt 2005 [30]	NR	NR	Peto OR	Lap 125/1650 (7.6%) Open 208/1642 (12.7%)	15 (3292)	0.58 (0.44, 0.70)			L	
Schmedt 2002 [31]	NR	NR	NR		8 (698)	NR			CL	8 studies reported more pain in open mesh group
*Laparoscopic vs Open non-mesh*										
Schmedt 2002 [31]	NR	NR	NR		6 (990)	NR			CL	Most studies reported less pain in laparoscopic group
*TAPP vs Open*										
McCormack 2003 [10]	All	Groin pain of any severity as near 12 months after the operation as possible provided this was at least after 3 months	Peto OR	Lap 150/1086 (13.8%) Open 227/1408 (16.1%)	15 (2494)	0·62 (0·49, 0·79)	7 (209)	1.00 (0.44, 2.25)	M	IPD
Aiolfi 2019 [15]	PH, UH	NR	RR	NR	NR	0.53 (0.27, 1.20)			H	NMA
O’Reilly 2012 [25]	PH, UH	Persistence of symptoms beyond 1 month	RR	NR	9 (2313)	0.66 (0.50, 0.87)			L	
*TAPP vs Open mesh*										
McCormack 2003 [10]	All	Groin pain of any severity as near 12 months after the operation as possible provided this was at least after 3 months	Peto OR	Lap 74/680 (10.9) Open 109/668 (16.3)	7 (1348)	0.59 [0.43, 0.83]	3 (153)	1.22 (0.49, 3.03)	M	IPD
Lyu 2020 [14]	All	NR	OR	NR	NR	0.51 (0.13, 1.7)			M	
Scheuermann 2017 [19]	PH	Persistent inguinal pain 3 months after surgery	OR	Lap 19/299 (6.4%) Open 37/284 (13.0%)	5 (583)	0.42 (0.23, 0.78)			M	Excluded irreducible and incarcerated hernia requiring emergency surgery
*TAPP vs Open non-mesh*										
McCormack 2003 [10]	All	Groin pain of any severity as near 12 months after the operation as possible provided this was at least after 3 months	Peto OR	Lap 48/618 (7.8%) Open 103/617 (16.7%)	8 (1235)	0.35 (0.24, 0.50)	2 (53)	0.18 (0.00, 9.42)	M	
*TEP vs Open*										
McCormack 2003 [10]	All	Groin pain of any severity as near 12 months after the operation as possible provided this was at least after 3 months	Peto OR	Lap 140/1015 (13.8%) Open 232/991 (23.4%)	6 (2006)	0·47 (0·36, 0·60)	2 (122)	0.80 (0.36, 1.81)	M	IPD
Aiolfi 2019 [15]	PH, UH	NR	RR	NR	NR	0.86 (0.48, 1.16)			H	NMA
O’Reilly 2012 [25]	PH, UH	Persistence of symptoms beyond 1 month	RR	NR	7 (2208)	0.81 (0.45, 1.44)			H	
*TEP vs Open mesh*										
McCormack 2003 [10]	All	Groin pain of any severity as near 12 months after the operation as possible provided this was at least after 3 months	Peto OR	Lap 2/193 (1.0%) Open 17/157 (10.8%)	3 (350)	0.13 (0.05, 0.34)	1 (36)	0.19 (0.01, 3.32)	M	IPD
Lyu 2020 [14]	All	NR	OR	NR	NR	0.85 (0.26, 2.0)			M	
Zheng 2014 [22]	All	Persistent groin pain or any groin discomfort affecting daily activities	RR	Lap 155/1368 (11.3%) Open 233/1458 (16.05)	9 (2826)	0.70 (0.59, 0.85)			L	
Koning 2013 [23]	PH	Persisting pain for longer than 3 months	RR	Lap 334/2692 (12.4%) Open 454/2705 (16.8%)	11 (5397)	0.80 (0.61, 1.04)			M	
Gavrilidis 2019 [17]	NR	Pain of any severity (including testicular) persisting for more than 3 months after the operation	Peto OR	Lap 178/1617 (11.0%) Open 219/1680 (13.0%)	13 (3479)	0.81 (0.66, 1.00)			M	
*TAPP vs Open non-mesh*										
McCormack 2003 [10]	All	Groin pain of any severity as near 12 months after the operation as possible provided this was at least after 3 months	Peto OR	Lap 13/498 (2.6%) Open 73/517 (14.1%)	2 (1015)	0.22 [0.14, 0.35]			M	IPD

^*^ H: high, M: moderate, L: low, CL: critically low

^#^ Review using qualitative synthesis and no meta-analysis

*All* includes both primary and recurrent inguinal hernias (PH and RH) and unilateral and bilateral inguinal hernias (UH and BH), *BH* Bilateral inguinal hernia, *CI* confidence interval, *CrI* credible interval, *IPD* used individual participant data, *Lap* laparoscopic, *NMA* network meta-analysis, *NR* not reported/no meta-analysis done, *OR* odds ratio, *Peto OR* Peto odds ratio, *PH* primary inguinal hernia, *RCT* randomised controlled trials, *RD* risk difference, *RH* recurrent inguinal hernia, *RR* relative risk, *TAPP* transabdominal pre-peritoneal repair, *TEP* totally extra-peritoneal repair, *UH* unilateral inguinal hernia

Most reviews of primary (or mixed primary/recurrent) hernias consistently suggested laparoscopic repairs to have a lower risk of chronic groin pain compared to open repairs, regardless of whether TAPP/TEP or open mesh/non-mesh was used, and although effect sizes varied, many reviews showed odds ratios around 0.5, indicating a 50% reduction in the odds of having chronic pain for laparoscopic repair compared to open repair (Table 4). In particular, in the four systematic reviews assessing any laparoscopic versus any open repairs, laparoscopic repair was associated with a statistically significant (range: 26% to 46%) reduction in the odds or risk of chronic pain. When considering the reviews of recurrent hernias alone, most studies did not show statistically significant results, but results were based on small number of trials [10, 20, 24, 28].

## Discussion

There was no clear evidence of a difference in recurrence rates between laparoscopic and open repairs for inguinal hernia in the systematic reviews and meta-analyses identified, although clinically important effects could not be ruled out due to the wide CIs reported. However, laparoscopic techniques were generally found to have less chronic groin pain compared to open repairs, regardless of the specific open or laparoscopic repair considered, or the hernia type (primary or recurrent hernia) that was studied. Overall, conclusions remained similar when examining either primary or recurrent inguinal hernias alone.

Reviews assessing recurrence rates showed heterogeneity in the magnitude and direction of effects. Most reviews which looked at primary hernias, recurrent hernias and both types of hernias together were rated high to moderate quality, suggesting high confidence in their findings. Other reviews which did not report the specific hernia types also had uncertain results with four reviews [8, 17, 26, 30] of moderate to low quality showing statistically significant results in favour of open repair techniques, but the generally low overall quality of these reviews remains a concern. These findings are similar to those published in a recent clinical guideline [33] in which experienced hernia surgeons and researchers across the world made recommendations based on comparable recurrence rates between laparoscopic and open repairs. The guideline emphasised that the recurrence rates remain comparable especially where the surgeries were conducted by highly skilled surgeons.

For chronic groin pain, we found that laparoscopic repairs were consistently associated with lower pain compared with open repairs regardless of the type of open or laparoscopic repair or the types of hernia studied. This is despite the noticeable difference in the definitions of chronic groin pain across the reviews we included. A recent review [18] reported both early and late chronic groin pain with distinct definitions, but the findings are similar and consistent with those of other reviews in this overview. Two reviews [20, 24] were also noted to have included exactly the same studies and participants in a number of comparisons of hernia types, although their findings are consistent with similar reviews.

In recurrent hernias, there are concerns about the risk of chronic pain, which is determined mainly by the chosen approach to repair. The HerniaSurge group guidelines [33] noted that repair of a recurrent hernia is always challenging compared with a primary hernia, and they emphasised that re-entry through a scar tissue increases risk of nerve and blood vessel entrapment/damage and potentially increases the risk of chronic groin pain and testicular atrophy. Therefore, the guideline recommends that if the prior repair is an open repair, then a laparoscopic approach is strongly favoured due to reduced risk of damage to structures.

The systematic reviews varied considerably in quality, included patients and outcome definitions. Attempts have been made to minimise the differences between reviews by categorising the review findings by hernia type and specific repair techniques to enable more meaningful interpretation of findings during the narrative synthesis. Because of the limitations of this narrative approach, a more robust synthesis, for example, a series of pairwise and network meta-analyses at different levels would be needed to provide comprehensive answers to research questions in this area including the type of mesh and the effect on different subgroups of patients.

This overview has a number of strengths including the pre-specification of methods in a protocol, which guided the conduct of the overview as recommended by the preferred reporting items for systematic reviews and meta-analysis (PRISMA) guidelines [12]. Study identification and selection were carried out by two researchers to enhance the integrity of the study selection process. Only systematic reviews and meta-analyses of RCTs were included in the overview to avoid the intrinsic bias of observational studies. Limitations of the overview include difficulty in retrieving some articles, exclusion of reviews published in languages other than English, restriction of outcome analysis to hernia recurrence and chronic groin pain, single data extraction and quality assessment and the exclusion of conference proceedings.

In conclusion, although the overview has found no clear evidence of differences in recurrence rates between laparoscopic and open repairs, laparoscopic techniques have generally been shown to have less postoperative long-term pain compared with open repairs.
